# Biomarkers Associated with Cardiovascular Disease in COVID-19

**DOI:** 10.3390/cells11060922

**Published:** 2022-03-08

**Authors:** Christoph C. Kaufmann, Amro Ahmed, Achim Leo Burger, Marie Muthspiel, Bernhard Jäger, Johann Wojta, Kurt Huber

**Affiliations:** 1 3rd Medical Department with Cardiology and Intensive Care Medicine, Klinik Ottakring (Wilhelminen Hospital), Montleartstrasse 37, 1160 Vienna, Austria; amro.ahmed@wienkav.at (A.A.); achim.burger@wienkav.at (A.L.B.); marie.muthspiel@extern.wienkav.at (M.M.); bernhard.jaeger@wienkav.at (B.J.); kurt.huber@meduniwien.ac.at (K.H.); 2 Medical School, Sigmund Freud University, 1090 Vienna, Austria; 3 Division of Cardiology, Department of Internal Medicine 2, Medical University of Vienna, 1090 Vienna, Austria; johann.wojta@meduniwien.ac.at; 4 Core Facilities, Medical University of Vienna, 1090 Vienna, Austria; 5 Ludwig Boltzmann Institute for Cardiovascular Research, 1020 Vienna, Austria

**Keywords:** COVID-19, cardiac biomarkers, myocardial injury, troponin, BNP

## Abstract

Coronavirus disease-19 (COVID-19) emerged late December 2019 in the city of Wuhan, China and has since spread rapidly all over the world causing a global pandemic. While the respiratory system is the primary target of disease manifestation, COVID-19 has been shown to also affect several other organs, making it a rather complex, multi-system disease. As such, cardiovascular involvement has been a topic of discussion since the beginning of the COVID-19 pandemic, primarily due to early reports of excessive myocardial injury in these patients. Treating physicians are faced with multiple challenges in the management and early triage of patients with COVID-19, as disease severity is highly variable ranging from an asymptomatic infection to critical cases rapidly deteriorating to intensive care treatment or even fatality. Laboratory biomarkers provide important prognostic information which can guide decision making in the emergency department, especially in patients with atypical presentations. Several cardiac biomarkers, most notably high-sensitive cardiac troponin (hs-cTn) and N-terminal pro-B-type natriuretic peptide (NT-proBNP), have emerged as valuable predictors of prognosis in patients with COVID-19. The purpose of this review was to offer a concise summary on prognostic cardiac biomarkers in COVID-19 and discuss whether routine measurements of these biomarkers are warranted upon hospital admission.

## 1. Introduction

Coronavirus disease-19 (COVID-19) emerged late December 2019, in the city of Wuhan, China, and has since spread internationally causing a global pandemic [1]. The disease is based on infection with severe acute respiratory syndrome coronavirus 2 (SARS-CoV-2), which commonly leads to the development of upper respiratory tract infection [2]. We have dealt with different coronaviruses in the past, including severe acute respiratory syndrome (SARS) and Middle East respiratory syndrome (MERS), the former originating in China with over 8000 cases and the latter being primarily reported in Saudi Arabia with over 2500 confirmed cases and a high fatality rate of up to 25% [3]. To date, a total of more than 340 million cases of COVID-19 with more than 5.5 million deaths have been reported worldwide, including more than 125 million confirmed cases in Europe alone [4].

**Figure 1:**
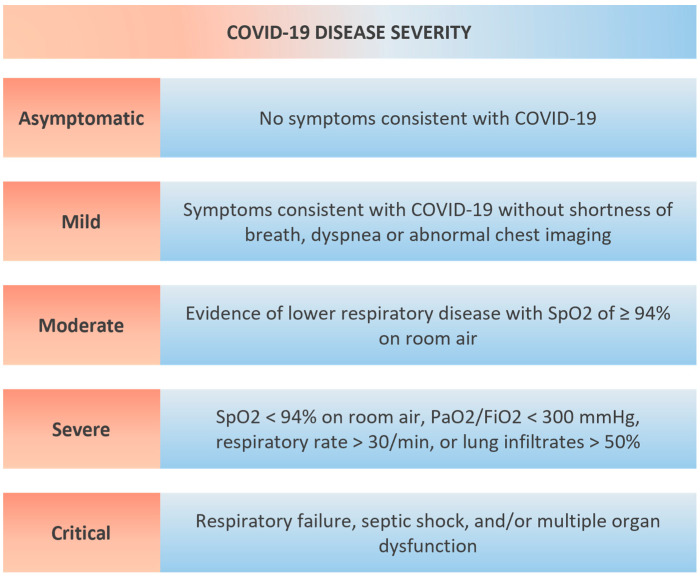
Severity of COVID-19. SpO2—oxygen saturation, PaO2/FiO2—arterial partial pressure of oxygen to fraction of inspired oxygen ratio.

Several SARS-CoV-2 variants have been described since the initial COVID-19 outbreak, which are defined by one or more of the following changes according to the World Health Organization (WHO): (A) Increase in transmissibility or detrimental change in COVID-19 epidemiology; (B) increase in virulence or change in clinical disease presentation; (C) decrease in effectiveness of public health and social measures or available diagnostics, vaccines, therapeutics. Currently, the WHO monitors several variants of concern, including Alpha (first document in the United Kingdom), Beta (first document in South Africa), Gamma (first document in Brazil), Delta (first document in India) and Omicron (first documented simultaneously in multiple countries) variants [5]. The clinical manifestation of COVID-19 is highly variable and can be classified as mild (variety of signs of symptoms without shortness of breath or abnormal chest imaging); moderate (evidence of lower respiratory disease with SpO2 ≥ 94% on room air); severe (SpO2 < 94% on room air, PaO2/FiO2 < 300 mmHg, respiratory rate >30 breaths/min or lung infiltrates >50%); or critical (respiratory failure, septic shock, and/or multiple organ dysfunction) (Figure 1) [6].

Early detection of patients at risk for deterioration has always been one of the main goals of laboratory biomarker research and as such has been carried out plentifully in patients with COVID-19. Among many others, several cardiac biomarkers have been identified that provide prognostic information concerning disease severity and clinical outcomes. Cardiovascular involvement in COVID-19 has been a topic of major discussion since the beginning of the pandemic, which originated from autopsy studies showing myocardial damage in these patients [7,8,9]. Additionally, an excess of myocardial injury, defined by an increase in high-sensitive cardiac troponin, has also been observed in COVID-19 alongside cases of myocarditis, acute right heart failure, thromboembolism, and arrhythmic complications up to cardiac arrest [10,11]. Recently, cardiac tropism was further underlined by the in situ labeling of SARS-CoV-2 ribonucleic acid (RNA) within cardiomyocytes, interstitial cells, and endothelial cells of postmortem hearts [12,13]. The direct pathophysiological link between COVID-19 and cardiovascular disease, however, has not been fully elucidated yet. Several theories trying to explain this association have emerged, including Angiotensin converting enzyme 2 (ACE2)-mediated injury, hypoxia-induced injury, microvascular thrombosis, and systemic inflammatory injury [14]. 

Vaccination rates for COVID-19 differ vastly according to country and continent, which may be explained by availability, governmental restrictions, and the general willingness of every single individual towards vaccination [15]. New SARS-CoV-2 variants with varying vaccine response rates have led to different vaccination approaches across the globe with booster vaccinations and different timings being a topic of controversy and ongoing discussion. Cardiovascular complications following SARS-CoV-2 vaccination have been reported with a predominance among otherwise often healthy young males [16,17]. Myocarditis, in particular, has been described throughout the literature as an adverse event, characterized by a significant increase in biomarkers of myocyte injury (most notably hs-cTn). Fortunately, symptoms of vaccine-related myocarditis have been largely described as modest and imaging findings upon echocardiography and cardiac magnetic resonance imaging are usually mild [18,19].

**Figure 2:**
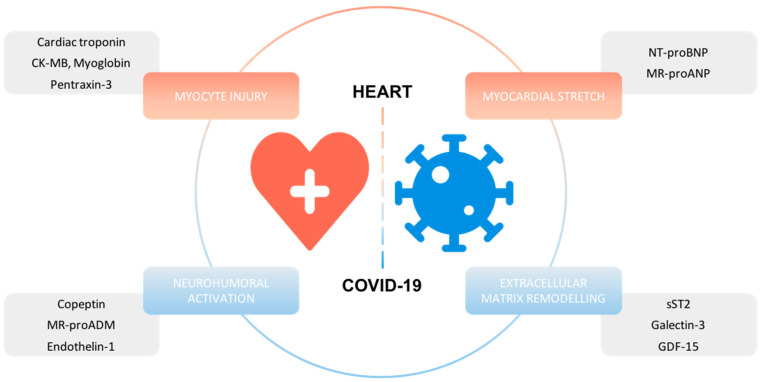
Overview of cardiac biomarkers with prognostic information in COVID-19.

The following review will summarize current evidence on prognostic cardiac biomarkers in patients with COVID-19 and discuss whether routine measurements of these biomarkers are warranted upon hospital admission (Figure 2, Table 1).

## 2. Biomarkers of Myocyte Injury

### 2.1. High-Sensitive Cardiac Troponin, Creatine Kinase and Myoglobin

Cardiac troponins are the pillars of biomarker research in acute cardiovascular care and have long been advocated by the international cardiac society for the diagnostic and prognostic assessment of acute coronary syndrome (ACS) [31,32]. While both high-sensitive cardiac troponin I (hs-cTnI) and troponin T (hs-cTnT) perform similarly in clinical practice, it is essential to be aware of different assays used throughout the literature to adequately translate research findings to your local protocol [33]. It is also important to remember that increases in hs-cTn can be rather unspecific, especially in the context of critical illness or infectious disease [34,35]. This is further illustrated by early findings of excess myocardial injury in hospitalized patients with COVID-19 with reported prevalence rates of 5–40% with myocardial injury being even more common among those with critical illness or a consequent fatal disease course [10,11,36,37]. The direct mechanism linking myocardial injury to SARS-CoV-2 remains elusive to this date. However, several theories have emerged that may explain this observed association, including direct effects on cardiac myocytes by the virus itself, Angiotensin enzyme 2 (ACE2)-mediated injury, hypoxia-induced injury, microvascular thrombosis, and systemic inflammatory injury [14].

Interpretation of hs-cTn levels in the emergency department can be challenging as ischemic cardiac causes have to be distinguished from bystander hs-cTn elevations in the context of COVID-19. Careful correlation to symptoms of ischemia, such as chest pain, as well as non-invasive work-up, including electrocardiogram (ECG) and echocardiography, may guide early decision making in these patients. Serial measurements of hs-cTn levels, alongside NT-proBNP, are important to establish peak values of these biomarkers and further increase clinical and prognostic information gained by biomarker analysis. The European Society of Cardiology (ESC) advocates different management strategies according to levels of hs-cTn and highlights that mild elevations in hs-cTn often rather reflect pre-existing cardiac disease—especially in the elderly—or acute injury related to COVID-19 and, hence, do not require urgent work-up or specific treatment [14]. However, management of patients with actual ACS should not be altered by co-infection with COVID-19 as early revascularization remains the top priority in these patients [38]. 

The prognostic value of hs-cTn in COVID-19 has been consistently shown in multiple, different study populations across the globe and was confirmed by data from several meta-analyses [39,40,41,42]. One of the more recent ones reported a significant association between COVID-19 disease severity and levels of cardiac troponin from pooled patient data of 28 studies comprising 7812 cases. These observations remained persistent irrespective of the troponin assay use, as significant associations were found for both hs-cTnI (standardized mean difference (SMD) = 0.66 pg/mL, 95% CI = 0.51–0.81, *p* < 0.001) and hs-cTnT (SMD, 0.93 U/L; 95% CI, 0.21–1.65; *p* = 0.012). Similar findings were reported for mortality prediction, which included data from 41 studies comprising 9532 cases. Hs-cTnI (SMD, 0.51 pg/mL, 95% CI, 0.38–0.63; *p* < 0.001) and hs-cTnT (SMD, 0.85 U/L; 95% CI, 0.63–1.07; *p* < 0.001) were associated with a significantly increased risk of overall mortality among patients with COVID-19 [43]. An area under the curve of 0.73 (0.69–0.77) was reported for the association between increased troponin and mortality in a different meta-analysis [44].

Several studies have also investigated the prognostic value of creatine kinase and myoglobin—often together with hs-cTn—in COVID-19 [45,46,47,48,49,50,51]. Pooled data from meta-analyses have shown a consistent increase in both creatine kinase–myoglobin binding (CK-MB) (SMD, 0.54 U/L; 95% CI, 0.39–0.69; *p* < 0.001) and myoglobin (SMD, 0.80 U/L; 95% CI, 0.57–1.03, *p* < 0.001) among patients with severe forms of COVID-19. This association also extended to mortality prediction for both biomarkers (CK-MB: [SMD, 0.48 U/L; 95% CI, 0.32–0.65; *p* < 0.001]; myoglobin: [SMD, 0.55 U/L; 95% CI, 0.45–0.65; *p* < 0.001]) [43].

### 2.2. Pentraxin-3

Pentraxin-3 (PTX3) is a member of the pentraxin superfamily and is similar in structure to C-reactive protein (CRP), which is homologous to the C-terminal domain of PTX3. Contrary to CRP, which is produced in the liver secondary to IL-6 stimulation, PTX3 is released as an acute phase protein in response to inflammation in different cell types, including macrophages, neutrophils, endothelial cells, and fibroblasts. The main stimuli for PTX3 release are IL-1 and tumor necrosis factor alpha [52,53]. Additionally, an association of PTX3 with myocardial injury following ischemia has been shown in animal studies and in humans with fatal myocardial infarction [54,55]. Besides infectious diseases, PTX3 has hence been studied extensively in cardiovascular disease and association with clinical outcomes was found in myocardial infarction, heart failure, and cardiac arrest [56].

An Italian cohort study on 96 patients hospitalized for acute COVID-19 was the first to investigate the prognostic value of PTX3 in this setting. The authors reported significantly higher PTX3 concentrations among patients with a fatal outcome and among those who required intensive care treatment during hospitalization. Upon multivariable Cox regression analysis, PTX3 was found to be an independent prognostic factor for short-term mortality (adjusted HR (aHR), 27.6; 95% CI, 5.3–142.8; *p* < 0.0001). These findings were validated in a second independent cohort of 54 patients from a different hospital. While baseline characteristics were vastly different between the two cohorts, the results remained consistent as PTX3 was also of prognostic value in this secondary analysis (crude HR, 6.9; 95% CI, 1.25–38.2; *p* = 0.026). In addition, this study also showed that PTX3 was primarily expressed by monocytes, lung macrophages, and endothelial cells, which further underlines the multi-system disease character of COVID-19 [57]. A relatively large biomarker study, including a total of 219 patients, measured PTX3 on a luminex assay—next to multiple other biomarkers—and also found a significant association with the primary endpoint of mortality regardless of whether patients were treated on a regular ward or in the intensive care unit (ICU) [58]. Since then, several smaller studies have further underlined the prognostic efficacy of PTX3 in COVID-19 [59,60,61,62]. 

An Italian study investigated the potential of a human monoclonal antibody, siltuximab, which inactivates IL-6 induced signaling, in a small cohort of 30 patients with critical COVID-19 requiring ventilatory support. The authors found that treatment with siltuximab resulted in a significant reduction in both IL-8 and PTX3, which was in turn associated with improved survival and ventilatory outcomes [63].

## 3. Biomarkers of Myocardial Stretch

### 3.1. BNP/NT-proBNP

Brain natriuretic peptides (BNP) are primarily secreted secondary to ventricular wall stress, which is usually caused by volume or pressure overload. Enzymatic cleavage of proBNP precipitates the release of the biologically active BNP and N-terminal pro-B-type natriuretic peptide (NT-proBNP) into the circulation, which is mediated by the proteolytic enzyme furin [64,65]. While both natriuretic peptides are released in equimolar amounts, higher levels of NT-proBNP can be observed in clinical practice, which may be attributed to the longer half-life time of NT-proBNP compared to BNP [66]. Owing to its pathophysiological properties, (NT-pro)BNP has been established as the main diagnostic and prognostic marker in heart failure. This extends to patients with reduced (HFrEF), mildly reduced (HFmrEF), and preserved ejection fraction (HFpEF). In addition to its efficacy in heart failure, (NT-pro)BNP has also been shown to improve outcome prediction in a variety of clinical conditions, including infectious and other cardiovascular diseases [67,68,69].

These findings were extended to patients with COVID-19, as numerous studies found significant associations between concentrations of (NT-pro)BNP upon hospital admission and subsequent clinical outcomes [70,71,72,73,74]. A representative single-center study from Spain included 396 COVID-19 patients presenting to the emergency department at a tertiary center during the first wave of the pandemic. While roughly half of the patients had NT-proBNP levels above the recommended cut-offs for the identification of heart failure (*n* = 192), only 47 fulfilled the clinical criteria of heart failure. The authors reported a significant association between NT-proBNP and mortality upon multivariable Cox regression analysis (HR 1.28 per logarithmic unit, 95% CI, 1.13–1.44; *p* < 0.001), which persisted after exclusion of patients with heart failure [75]. Data from meta-analyses further underlined the prognostic value of both BNP and NT-proBNP with regard to mortality and disease severity in patients with COVID-19 [42,76,77,78]. While the direct causal relationship between COVID-19 and natriuretic peptides have not been elucidated yet, many theories have been suggested to explain this association. Myocardial injury, inflammation itself, interaction with Angiotensin converting enzyme (ACE2), or impairments of cardiac function with acute heart failure may be responsible for higher circulating natriuretic peptides in COVID-19 [75]. 

### 3.2. MR-proANP

Atrial natriuretic peptide (ANP) is primarily secreted secondary to atrial volume or pressure overload and has important regulatory functions in natriuresis, diuresis and vasodilation [79]. Since the stability of ANP is rather poor ex vivo, the mid regional fragment of the precursor hormone of ANP—mid-regional pro atrial natriuretic peptide (MR-proANP)—has been established as a good and stable marker of ANP activity [80]. MR-proANP has been predominantly studied in the context of both acute and chronic heart failure and serves as a diagnostic and prognostic biomarker in both entities. In addition, several studies have also underlined the prognostic utility of MR-proANP in a variety of other clinical conditions, including respiratory, infectious, and other cardiovascular diseases [81,82,83,84].

To date, the prognostic value of MR-proANP has only been studied in an Austrian cohort of hospitalized patients with COVID-19 in a prospective, observational study led by our research group. We found significantly higher levels of MR-proANP among those with a fatal outcome within the follow-up period of 28 days compared to survivors (median, 307 pmol/L [IQR, 161–532] vs. median, 75 pmol/L [IQR, 43–153], *p* < 0.001). A significant correlation with disease severity, assessed by the Pneumonia Severity Index, and hypoxemia was also shown within our study. MR-proANP was a significant, independent predictor of 28-day mortality, even after adjustment for clinical confounders, comorbidities, and established prognostic markers for COVID-19 (HR, 2.77; 95% CI, 1.21–6.37; *p* = 0.016). The prognostic value of MR-proANP was numerically higher compared to NT-proBNP and we also showed the predictive potential of hs-cTnI for clinical outcomes in our study population. Further research data on MR-proANP is necessary to further characterize these observations. There are several pathophysiological links that may contribute to the association of MR-proANP with COVID-19. Most of these mechanisms are closely linked to pathophysiological considerations with NT-proBNP, since both biomarkers are secreted upon volume overload, one primarily by ventricular and the other by atrial myocytes [85].

## 4. Biomarkers of Extracellular Matrix Remodeling

### 4.1. SST2

ST2 is a member of the interleukin 1 (IL-1) family and is often referred to as interleukin 1 receptor-like 1. There are two isoforms of ST2—the soluble form (sST2) and the transmembrane receptor (ST2L)—that have to be discussed separately upon determination of the prognostic value of this biomarker. ST2L acts as the receptor for IL-33 and the interaction of the two has been shown to elicit cardioprotective effects with attenuation of cardiac fibrosis, hypertrophy, and apoptosis. SST2, however, actively binds to IL-33 and hence competes with the ST2L / IL-33 interaction, which in turn reduces the aforementioned cardioprotective effects. SST2 can be measured reliably in human serum by commercially available ELISA kits [86,87,88]. Owing to its pathophysiological involvement in several pathways of cardiovascular disease, sST2 has been shown to be of value in the prognostic evaluation of patients with acute cardiovascular disease. Since ST2 is closely linked to myocardial remodeling, it has been used predominantly in heart failure as a marker of risk [89].

SST2 was first explored in a Chinese study population of 80 hospitalized patients with COVID-19 of varying disease severity. The authors made two major observations: a significant increase in sST2 in patients with severe COVID-19 compared to those with a mild disease course and higher levels of sST2 in patients with COVID-19 compared to healthy controls. Upon multivariable logistic regression, this resulted in a significant association between sST2 and short-term mortality (OR, 5.876; 95% CI, 2.737–9.211; *p* = 0.003). A significant correlation between sST2 and markers of inflammation, including both CRP and procalcitonin, was also observed [90]. These findings were further strengthened by a cohort study from Spain on 152 hospitalized patients with COVID-19, which reported a significant increase in ICU admission or death among those with sST2 levels above 58.9 ng/L. They also showed that sST2 peak concentrations were reached 48-72 h after admission [91]. Additional research data have reported an association between higher levels of sST2 and an increased risk of invasive ventilation, as well as a tendency of sST2 to decline upon recovery in patients with a benign clinical course [92,93].

### 4.2. Galectin-3

To date, fifteen different galectins have been described throughout the literature with galectin-3 gaining traction as a biomarker of risk in patients with acute cardiovascular disease. Galectin-3 differs from the other galectins as it has a unique chimeric, biochemical structure, which makes it the only galectin with the ability to form pentamers [94]. It can be found in several organ systems of the human body and is widely distributed in the heart, lungs, and kidneys. Galectin-3 is primarily secreted by macrophages and fibroblasts secondary to acute inflammatory processes. In addition, it has also been linked to angiogenesis and endothelial damage [95,96]. The multisystem facete of the biomarker makes it an interesting target in several clinical conditions, including cardiovascular, renal, and pulmonary disease [97,98,99].

Galectin-3 has been studied recently in an Italian cohort of hospitalized COVID-19 patients with acute respiratory failure (*n* = 156). Significantly higher concentrations of galectin-3 were found in those with a fatal event within the follow-up period of 30 days (median, 43.8 ng/mL; IQR, 36.2–59 versus median, 21.9 ng/mL; IQR, 17.6–27.5; *p* < 0.001). After multivariable Cox regression analysis, galectin-3 remained a significant predictor of mortality alongside other biomarkers, such as IL-6 and CRP. The authors also reported that higher levels of galectin-3 were associated with admission to the ICU and a higher risk of acute respiratory distress syndrome (ARDS) [100]. A Turkish study extended these findings by showing that galectin-3 levels were significantly increased among patients with severe COVID-19 compared to non-severe cases and that galectin-3 levels were higher in patients with COVID-19 compared to healthy controls (median of severe COVID-19, 415.31 pg/mL; IQR, 122.81–1622.23 vs. median of non-severe, 326.33 pg/mL; IQR, 100.09–1271.04 vs. median of healthy controls 243.13 pg/mL, 166.57–380.41) [101]. Higher levels of galectin-3 among COVID-19 patients were also reported in a rather small Taiwanese study [102].

### 4.3. GDF-15

Growth differentiation factor 15 (GDF-15) is an inflammatory biomarker and a member of the transforming growth factor ß superfamily. As such, it is widely distributed throughout most organ systems of the human body [103]. Macrophages, endothelial cells, and cardiomyocytes have been shown to increase their production and secretion of GDF- 15 during oxidative stress, tissue injury, or inflammation. The effects of GDF-15 have not been fully understood yet, but several reports have pointed towards cardioprotective effects [104,105]. GDF-15 has been established as a powerful biomarker across different entities of cardiovascular disease, with a special focus on heart failure attributed to its consistent prognostic value in both preserved and reduced ejection fraction [106,107]. Moreover, several studies have shown a significant increase in GDF-15 in patients with inflammatory disease, which resulted in an increased risk of both morbidity and mortality among those with higher levels of GDF-15 [108,109].

Similarly, GDF-15 has also been studied in COVID-19, where it was successfully proven to be of predictive value for important clinical outcomes. A Norwegian study measured levels of GDF-15 in 123 hospitalized patients with COVID-19 and found significantly higher concentrations among those with a primary endpoint of admission to the ICU or death during hospitalization (median, 4225 pg/mL; IQR, 3197–5972 versus median, 2187 pg/mL; IQR, 1344–3620; *p* < 0.001). This association persisted after adjustment for clinical comorbidities and other selected biomarkers of interest (IL-6, CRP, procalcitonin, ferritin, D-dimer, troponin T and NT-proBNP). The authors also reported a correlation between GDF-15 and detectable SARS-CoV-2 viremia as well as hypoxemia in their study population [110]. The first study to investigate GDF-15 in COVID-19 was a hypothesis-generating case series of 66 patients, which showed an increase in GDF-15 in COVID-19 cases with a fatal outcome. These findings were confirmed after adjustment for the sepsis-related organ failure assessment (SOFA) score, a well-validated score in intensive care medicine [111]. Other studies with smaller patient cohorts have also reported an association between GDF-15 and COVID-19 severity, with dynamic changes in GDF-15 being closely associated with disease progression; in addition, a significant increase in GDF-15 among patients with COVID-19-related ARDS treated in the ICU compared to healthy controls was reported, with rising GDF-15 levels among non-survivors and decreasing levels among survivors [111,112,113,114].

## 5. Biomarkers of Neurohumoral Activation

### 5.1. Copeptin

The peptide neurohormone arginine vasopressin has an important role in the regulation of fluid balance as it decreases water excretion and increases urine osmolarity in the kidneys by inducing water absorption in the renal tubules and by vasoconstriction [115]. It is primarily produced within the hypothalamus and is secreted secondary to hyperosmolarity, hypotension, and stress [116]. Since the stability of arginine vasopressin is rather limited ex vivo, copeptin can be measured as a surrogate marker of vasopressin activity as it is secreted in equimolar amounts [117]. Copeptin has been shown to be of prognostic value in several respiratory and inflammatory conditions, including community-acquired pneumonia, ventilator-associated pneumonia, pulmonary embolism, and chronic obstructive lung disease [118,119,120,121]. A dual biomarker approach using both copeptin and cardiac troponin for the rapid rule-out of myocardial infarction was shown to be superior to standard single biomarker rule-out with cardiac troponin—research on high-sensitive cardiac troponins is still underway [122]. Recently, copeptin has also been investigated in patients with type II myocardial infarction with promising results—a clinical entity in desperate need of further research [123].

Our research group has studied copeptin in 213 hospitalized patients with COVID-19 in a prospective, observational study. Median levels of copeptin were significantly higher in patients with an adverse outcome (29.6 pmol/L; IQR, 16.2–77.8 vs. 17.2 pmol/L; IQR, 7.4–41.0; *p* < 0.001). A dual marker approach using both copeptin and hs-cTnI resulted in an independent significant association with the primary endpoint of ICU admission or death within 28 days (OR, 4.274; 95% CI, 1.995–9.154; *p* < 0.001). The addition of both biomarkers to clinically established risk scores in COVID-19 (e.g., 4C Clinical deterioration index) improved C-statistics and net reclassification indices, suggesting incremental prognostic information is provided by this dual biomarker strategy upon admission. Our study also showed that patients with increased copeptin and hs-cTnI were more likely to have ECG abnormalities, such as atrial fibrillation, bundle branch block, or low voltage [124]. A study from Switzerland also showed a significant association between copeptin and short-term mortality in a smaller study population of 74 patients. In addition, they found that the pattern of prognostic information provided by copeptin was similar across different respiratory infections (COVID-19 vs. non-COVID-19 pneumonia vs. acute/chronic bronchitis) [125]. A third study in 90 hospitalized patients with COVID-19 identified copeptin as a potential predictor for severity of COVID-19, which further adds to the clinical applicability of the biomarker [126].

### 5.2. MR-proADM

Adrenomedullin (ADM) is a peptide hormone that belongs to the calcitonin gene-related peptide family and was first isolated from phaeochromocytoma tissue at the end of the 20th century [127]. Widespread use of ADM as a clinical biomarker was initially limited by insufficient in vitro stability, which was solved by the introduction of mid-regional (MR) pro-ADM. It represents a stable fragment of ADM that can be used as a surrogate marker of ADM activity [128]. The main physiological effects of ADM are diuresis, natriuresis, and vasodilatation, which make ADM a biomarker of particular interest in patients with heart failure, where excess levels of ADM have been described repeatedly [129,130]. Some studies suggested a superior outcome prediction using MR-proADM compared to commonly used biomarkers, such as natriuretic peptides, in patients with both acute and chronic heart failure [131,132]. The use of MR-proADM for risk prediction has also been extended to sepsis and other infectious diseases, where MR-proADM has been shown to predict both morbidity and mortality [133,134]. 

These findings led to numerous studies assessing the prognostic value of MR-proADM in patients with COVID-19. The first of those studies investigated MR-proADM in a small cohort of hospitalized patients (*n* = 89) at a tertiary care center in Switzerland during the first COVID-19 wave in 2020. A 1.5-fold increase in median admission levels of MR-proADM were found among non-survivors compared to survivors (1.3 pg/mL; IQR, 1.1–2.3 vs. 0.8 pg/mL; IQR 0.7–1.1), which resulted in a significant independent association between MR-proADM and the primary endpoint of in-hospital mortality (adjusted odds ratio of 5.5; 95% CI, 1.4–21.4; *p* = 0.015) [135]. The largest study to date with a study population of 359 hospitalized COVID-19 patients showed similar results, with a significant association between MR-proADM and mid-term mortality of 90 days. The authors also found significantly higher levels of the biomarker among patients with a higher SOFA score [136]. The role of MR-proADM as a marker of prognosis in patients with COVID-19 has been confirmed by several further studies, both in patients with critical illness and among those on regular wards of the hospital [137,138,139,140,141,142,143,144,145,146,147].

### 5.3. Endothelin-1

Endothelin-1 (ET-1) is a 21-amino-acid peptide that is primarily synthesized and secreted by endothelial cells [148]. It plays a key role in the regulation of vasculature as it exerts both vasoconstriction through the endothelin receptor A and vasodilatation by induction of nitric oxide synthesis through the interaction with endothelin receptor B. Additionally, ET-1 has been shown to increase secondary to acute inflammation, which is precipitated by increased wall stress to the endothelium [149,150]. Due to its vascular effects, ET-1 has been linked closely to arterial hypertension, while also being of prognostic value across several other cardiovascular disease conditions, including coronary heart disease and heart failure [151]. Since endothelial dysfunction has been shown to contribute to the pathophysiology of COVID-19 and cardiovascular involvement, ET-1—a strong marker of endothelitis—has been suggested to provide prognostic information in these patients [152,153].

To date, conflicting results have been published concerning the prognostic impact of endothelin-1 in COVID-19. A small Swiss study measured endothelin-1 precursor peptide (proET-1) in 74 hospitalized patients with COVID-19 and, while numerically higher levels of proET-1 were reported in non-survivors (median, 81.8 pmol/L; IQR, 76 to 118 vs. median, 53.6; IQR, 37 to 69), no significant association with mortality was found upon multivariable logistic regression. Concentrations of proET-1 were also significantly lower compared to patients with non-COVID-19-associated pneumonia or exacerbated bronchitis [154]. Contrary to these findings, C-terminal proendothelin-1 was found to be of prognostic value in a second study of critically ill patients with confirmed SARS-CoV-2 pneumonia. The study population was slightly larger with 105 patients and consisted of sicker patients, which contributes to a higher number of events. C-terminal proendthelin-1 levels ≥111 pmol/L were significantly associated with the primary endpoint of 28-day mortality after multivariable adjustment (HR, 3.72; 95% CI, 1.71–8.08; *p* = 0.01) [140]. Considering the published evidence on endothelin-1 in COVID-19, we cannot draw a definite conclusion with regard to the prognostic impact of this biomarker—further research is needed to answer this question.

## 6. Other Biomarkers

### Osteopontin/(A)Symetric Dimethlyarginine (ADMA/SDMA)/Myeloperoxidase

Osteopontin (OPN) is a phosphorylated glyophosphoprotein that is involved in several pathophysiological pathways of the human body through the regulation of inflammatory cells [155]. The prognostic value of circulating OPN has been studied in a sizeable cohort of 341 hospitalized COVID-19 patients in the United States of America, Germany, and Greece. The authors found significantly higher levels of serum OPN in patients with COVID-19 compared to healthy volunteers. After multivariable analyses, OPN was significantly associated with mortality and the risk of mechanical ventilation [156]. Further studies have underlined the value of OPN by showing a significant association with COVID-19 disease severity and a direct relationship with inflammatory markers [157,158].

Asymmetric dimethylarginine (ADMA) is an endogenous inhibitor of nitric oxide synthase and has been linked to endothelial dysfunction and the progression of atherosclerosis [159]. A small study of 31 hospitalized patients with COVID-19 showed that concentrations of ADMA and symmetric DMA were significantly higher in those with a fatal disease course. The authors suggested that sequential analysis of both biomarkers allowed risk discrimination by categorizing patients into high risk, intermediate risk, or low risk according to biomarker analysis. These interesting findings need to be confirmed in a larger patient population [160].

Myeloperoxidase (MPO) is an enzyme found in neutrophil granulocytes and monocytes. It is an important part of the natural immune defense as it modulates the process of phagocytosis [161]. As such, MPO has been studied extensively as a marker of inflammation in COVID-19. Alongside other inflammatory markers, levels of MPO have been shown to increase in patients with COVID-19 compared to healthy controls [162]. MPO was also associated with disease severity and viral RNA load of SARS-CoV-2 [163,164,165].

## 7. Routine Measurement of Cardiac Biomarker in COVID-19?

The routine measurement of cardiac biomarkers in patients with COVID-19 has been a major topic of discussion since the beginning of the pandemic. Several advantages and disadvantages have to be considered:

1. While cardiac biomarkers have been proven to provide prognostic information in patients with COVID-19, questions remain about the incremental prognostic value upon addition of these biomarkers to established clinical risk models.
2. Asymptomatic increases in biomarkers (e.g., hs-cTn or NT-proBNP) may lead to unnecessary diagnostic work-up and may pose the involved health care personnel to an additional risk of infection.
3. In case of myocardial injury, a clear correlation to symptoms and signs of myocardial ischemia (chest pain, ECG and echocardiogram) is necessary to appropriately guide management decisions. If myocardial ischemia is found, patients should be treated as acute coronary syndromes in line with current guidelines with a special focus on the safety of associated health care personnel.
4. Future research on the potential pathophysiological mechanisms linking cardiovascular disease to COVID-19 is necessary to further define and improve knowledge on the prognostic value of cardiac biomarkers in these patients.
5. There is a significant lack of direct comparisons of cardiac biomarkers in patients with COVID-19, which is a necessity to ultimately define the “best” biomarker for risk prediction in this patient population.

While the Study Group on Biomarkers in Cardiology of the Acute Cardiovascular Care Association of the ESC has acknowledged the prognostic information gained by measurement of cardiac biomarkers in patients with COVID-19, an ultimate decision for biomarker testing in these patients should be made by the treating physician with careful consideration of the individual patient profile [166]. Further research data are necessary to obtain more information on pathophysiological associations and to ultimately establish the most efficient cardiovascular biomarker in COVID-19.

## Figures and Tables

**Table 1:** Characterization of Cardiac Biomarkers by Primary Origin, Release Mode, Normal Range and Mortality Prediction in COVID-19.

Biomarkers	Primary Origin	Primary Release Mode	Molecular Weight (kD)	Normal Range	Mortality in COVID-19
Biomarkers of Myocyte Injury					
Hs-cTn	Cardiac myocytes	Myocardial injury	24 (Trop I) 35 (Trop T)	Assay specific cut-offs	I
CK-MB	Cardiac myocytes, skeletal muscle	Muscle injury (primarily heart)	84	5–25 IU/L [20]	I
Myoglobin	Cardiac myocytes, skeletal muscle	Muscle injury	17.7	Mean 31 ng/mL (+/− 1.3) [21]	I
Pentraxin-3	Smooth muscle cells, vascular endothelial cells, macrophages	Vascular injury/inflammation	40.2	M: 1.87 ng/mL (CI, 1.81–1.94) [22] F: 2.12 ng/mL (CI, 2.05–2.19) [22]	I
Biomarkers of Myocardial Stretch					
NT-proBNP	Ventricular myocytes	Ventricular volume or pressure overload	8.5	M: 42.5–106.4 pg/mL (97.5 percentile) [23] F: 111.0–215.9 pg/mL (97.5 percentile) [23]	I
MR-proANP	Atrial myocytes	Atrial volume or pressure overload	2.6 (ANP)	M: 90.2–228 pmol/mL (97.5 percentile) [24] F: 118–214 pmol/mL (97.5 percentile) [24]	III
Biomarkers of Extracellular Matrix Remodeling					
sST2	Cardiac and lung cells	Inflammation/fibrosis/vascular congestion	37	M: mean 24.9 ng/mL (95% CI, 23.7–26.2) [25] F: mean 16.9 ng/mL (95% CI, 16.1–17.7) [25]	II
Galectin-3	Mostly non-cardiac: macrophages/ fibroblasts	Fibrosis	30	Median 62 ng/mL (IQR, 20–313) [26]	II
GDF-15	Unknown	Inflammation	16.7	2.5 percentile: 399 ng/L (90% CI, 399–399) [27] 97.5 percentile: 1335 ng/L (90% CI, 1152–1445) [27]	I
Biomarkers of Neurohumoral Activation					
Copeptin	Pituitary gland	Osmotic stimulation/stress	4021	Median 4.2 pmol/L (IQR, 1.0–13.8) [28]	II
MR-proADM	Ubiquitary	Neurohumoral activation/inflammation	5.1	2.5 percentile: 0.21 nmol/L (90% CI, 0.19–0.23) [29] 97.5 percentile: 0.57 nmol/L (90% CI, 0.55–0.59) [29]	I
Endothelin-1	Vascular endothelial cells	Pleiotropic (pulsatile stretch, hypoxemia…)	2492	M: 2.64 ng/L (IQR, 2.21–3.17) [30] F: 2.46 ng/L (IQR, 2.05–2.94) [30]	III

I—more than 3 studies with mortality prediction in COVID-19; II—2 or 3 studies with mortality prediction in COVID-19; III—less than 2 studies with mortality prediction in COVID-19; kD—kilodaltons; CI—confidence interval; IQR—interquartile range; Hs-cTn—high-sensitive cardiac troponin; CK-MB—creatine kinase–myoglobin binding; NT-ptoBNP—N-terminal pro-B-type natriuretic peptide; MR-proANP—Mid-regional pro atrial natriuretic peptide; sST-2—soluble ST2; GDF-15—growth differentiation factor 15; MR-proADM—mid-regional adrenomedullin.

## Data Availability

Not applicable.
